# Supplementary material for: In Vitro and In Vivo Evaluation of 6-O-α-Maltosyl-β-Cyclodextrin as a Potential Therapeutic Agent Against Niemann-Pick Disease Type C
Source: Int J Mol Sci. 2019 Mar 6;20(5):1152. doi: 10.3390/ijms20051152 (PMC6429330; doi:10.3390/ijms20051152)
Supplement: Supplementary file 1 [file ijms-20-01152-s001.pdf]

Supplementary Figures

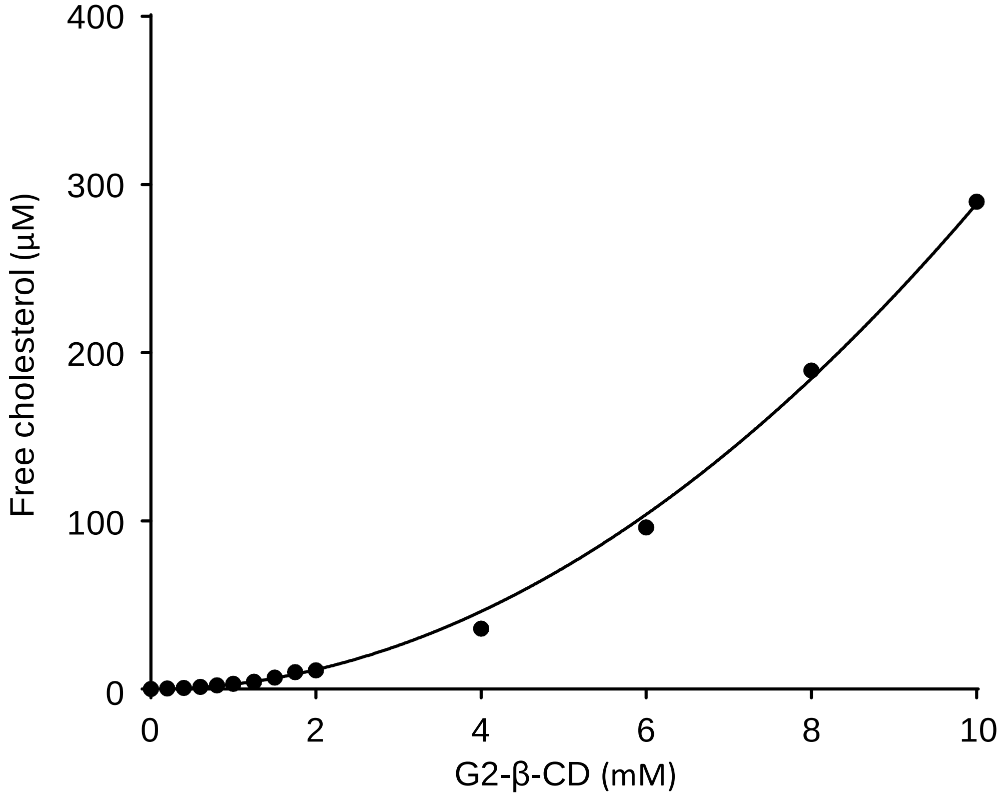

Supplementary Figure S1. Solubility-curve analysis to evaluate the solubilizing ability of G2-β-CD (closed circle) with FC in culture media. The solubility of FC was measured in DMEM/F12 medium (1:1) at 37°C. Each point represents the mean of 2 determinations.

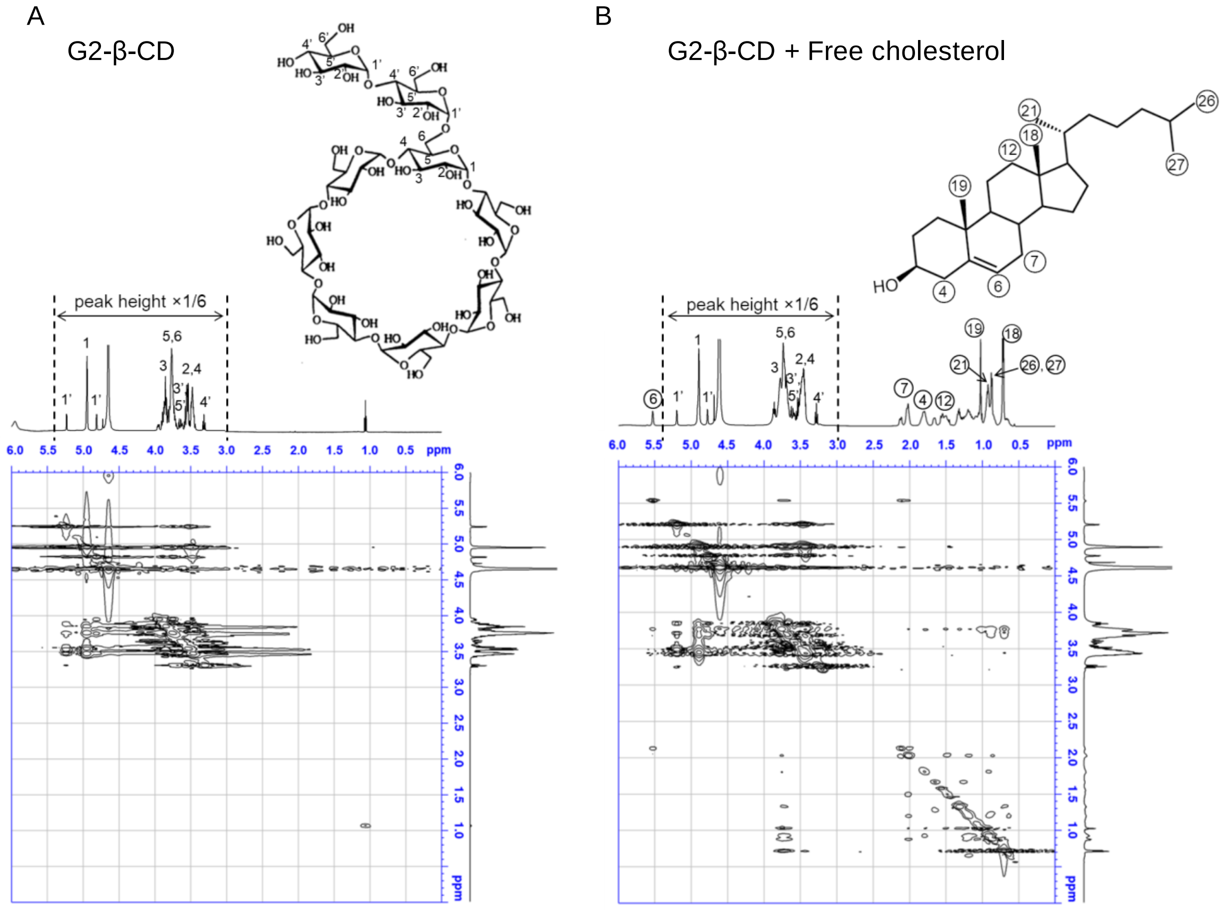

Supplementary Figure S2. Two-dimensional 1H-NMR ROESY spectrum of the G2-β-CD solution (A) and the G2-β-CD and FC solution (B). G2-β-CD (100 mM) and FC was dissolved in D2O.

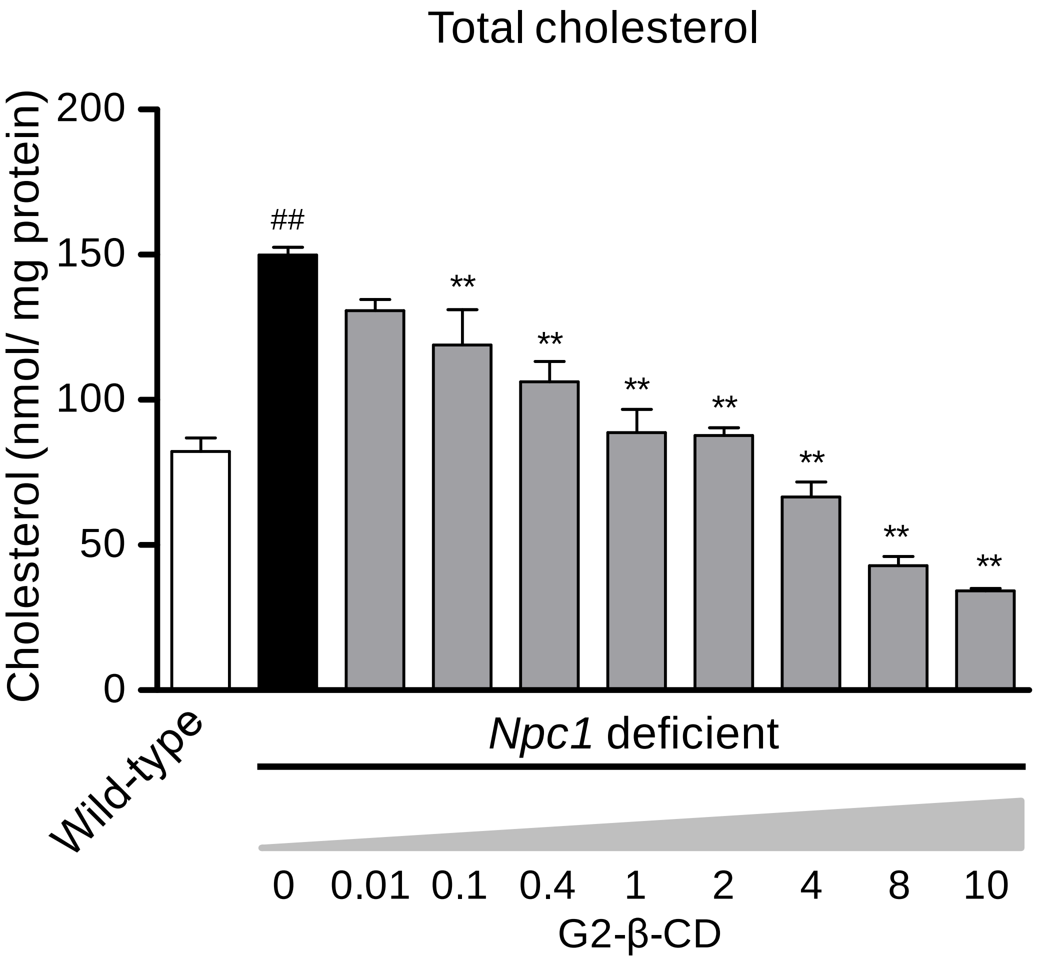

Supplementary Figure S3. Concentration-dependent effects of G2-β-CD on intracellular total cholesterol (TC) levels in *Npc1* deficient cells. TC levels were measured 24 h after the G2-β-CD treatment of *Npc1* deficient cells. Each bar represents the mean ± S.E.M. (n = 6). ##P < 0.01 compared with the Wild-type group. **P < 0.01 compared with the 0 mM group.

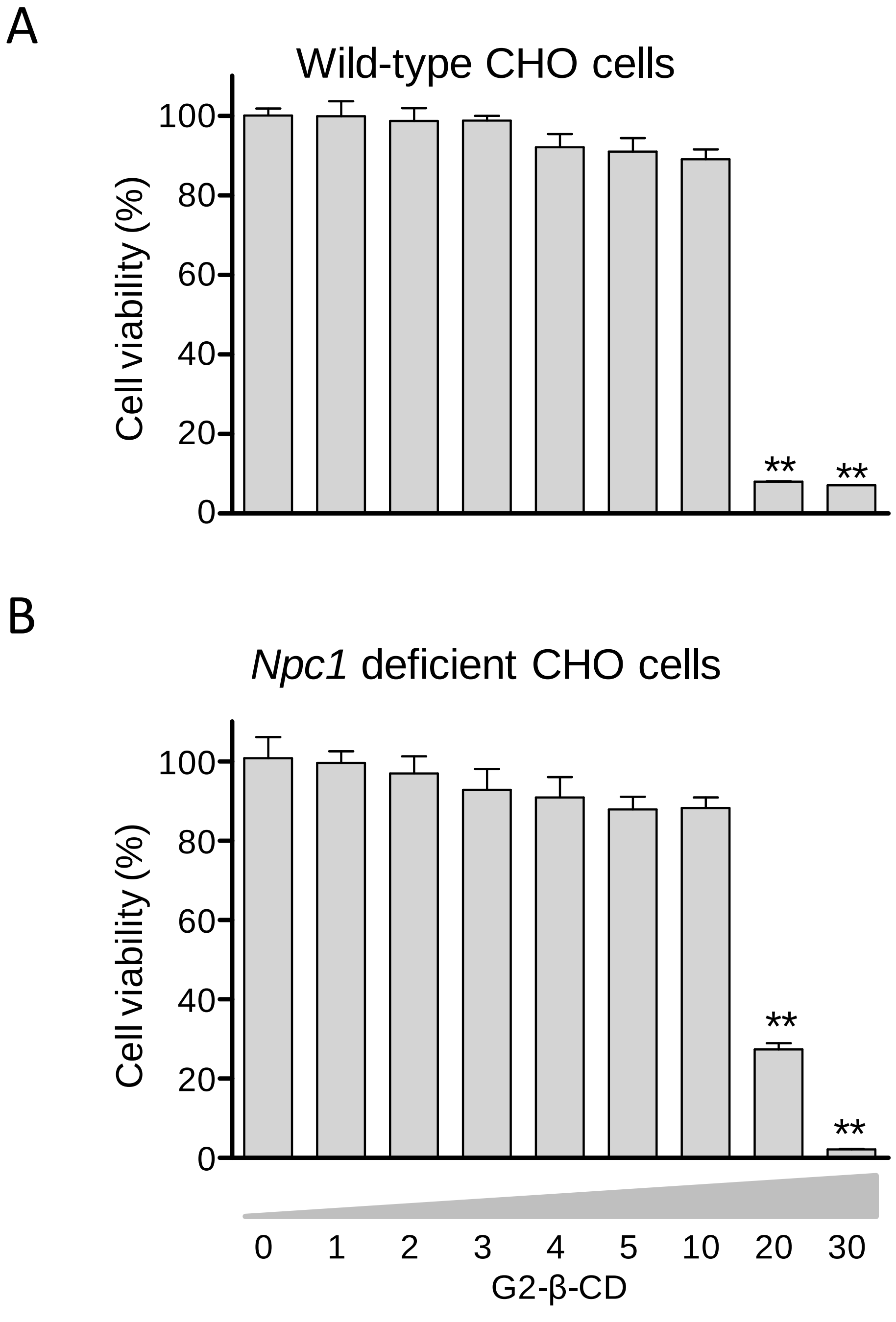

Supplementary Figure S4. Cytotoxicity evaluation of G2-β-CD in Wild-type and *Npc1* deficient CHO cells. Cell viability was observed 24 h after G2-β-CD treatment using the WST-8 assay. Each bar represents the mean ± S.E.M (n=4-6). **P < 0.01 compared with the 0 mM group.

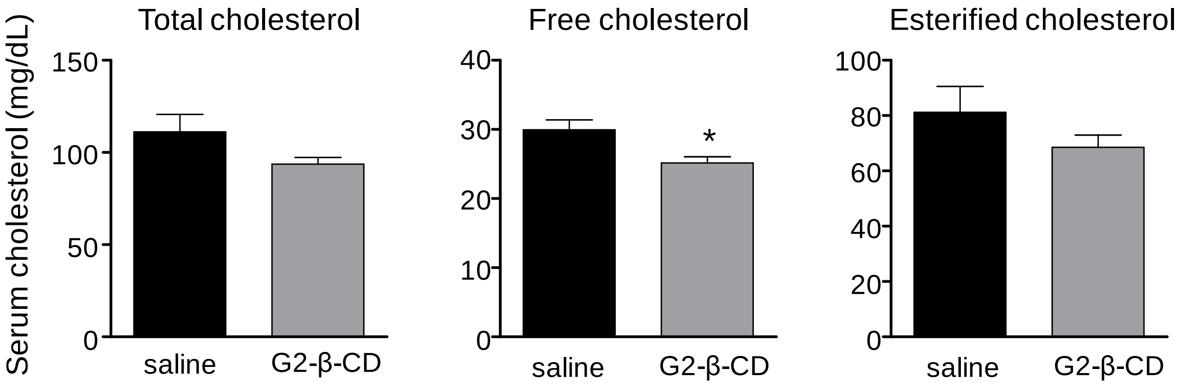

Supplementary Figure S5. Effect of systemic G2-β-CD treatment on serum cholesterol in *Npc1*$^{-/-}$ mice. Mice were subcutaneously treated with G2-β-CD (2.9 mmol/kg) at 6 weeks of age and serum samples were collected 72 h after the administration (n = 3). *P < 0.05 compared with the saline group.

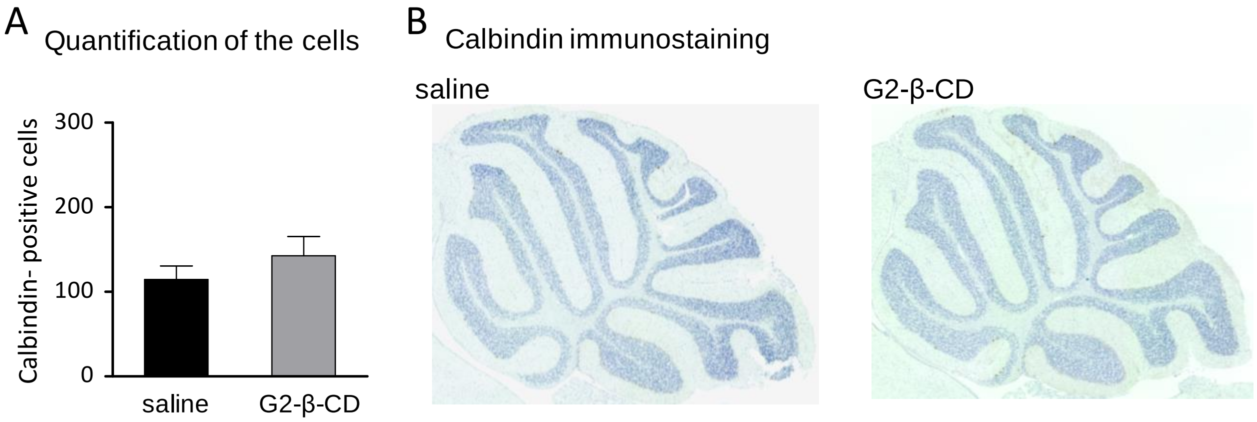

Supplementary Figure S6. Effects of subcutaneous injection of G2-β-CD on Purkinje cell loss in *Npc1*$^{-/-}$ mouse brain. Mice were subcutaneously treated with G2-β-CD (2.9 mmol/ kg) from 6 to 8 weeks of age, and then serum and brain samples were collected at 8 weeks and 2 days of age. Immunohistochemical staining of calbindin was performed in the mouse brain. The number of calbindin-positive cells in the cerebellum was measured (A). Representative images are shown (B). Each point and bar represents the mean ± S.E.M. (n = 6).

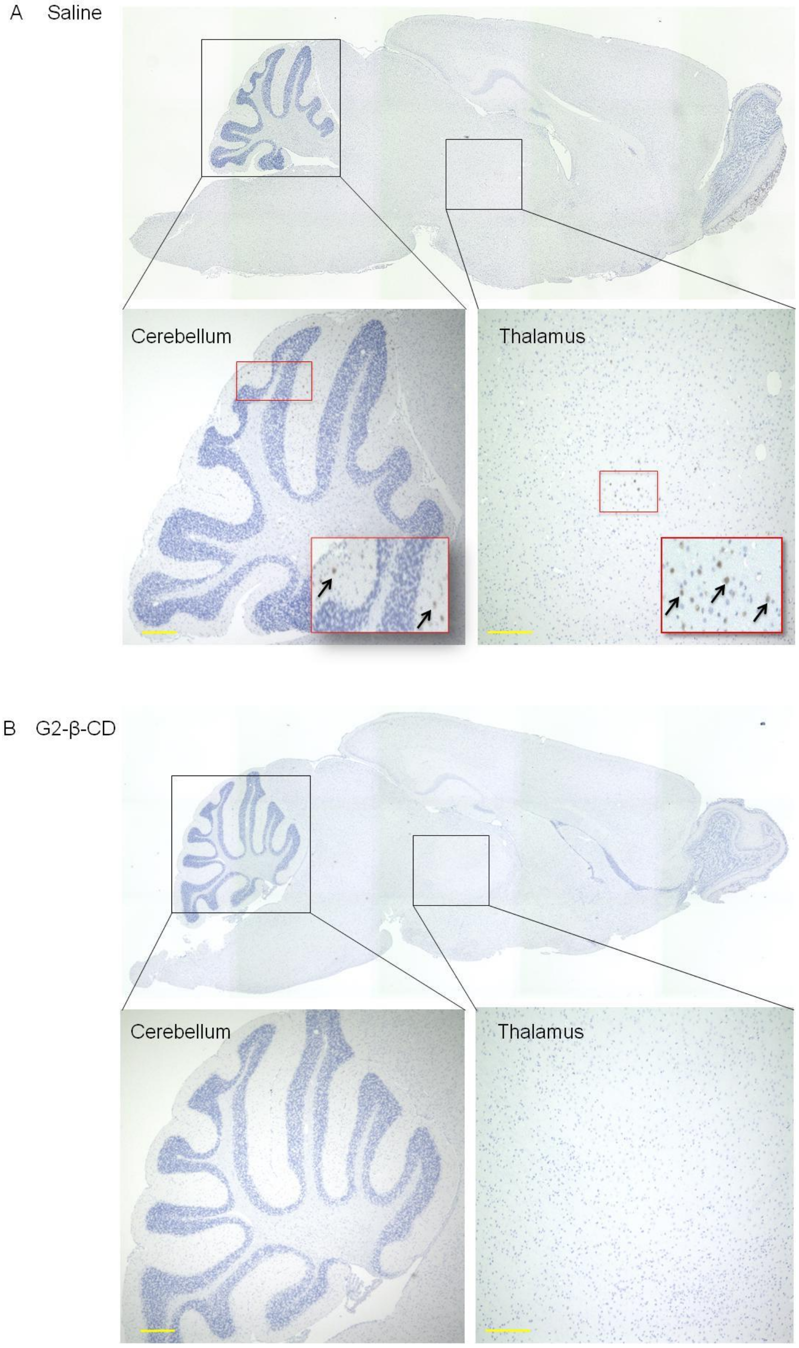

Supplementary Figure S7. Effects of an intracerebroventricular injection of G2-β-CD on osteoactivin/glycoprotein non-metastatic protein B (GPNMB) in *Npc1*$^{-/-}$ mouse brain. An injection of G2-β-CD (21.4 μmol/kg) was performed in mice at 4 weeks of age. A brain sample was collected at 8 weeks and 2 days of age and immunohistochemical staining of GPNMB was performed. Representative images of saline (A) and G2-β-CD (B)-treated groups are shown. Upper: whole brain, lower left: cerebellum, lower right: thalamus Scale bar: 200 μm.
